# A cross sectional study of leptospirosis and fetal death in Yucatan, Mexico

**Published:** 2016-03-30

**Authors:** María Fidelia Cárdenas-Marrufo, Ignacio Vado-Solis, Carlos Pérez-Osorio, Gaspar Peniche-Lara, José Segura-Correa

**Affiliations:** 1 Laboratorio de Enfermedades Infecciosas y Parasitarias II. Facultad de Medicina de la Universidad Autónoma de Yucatán. Merida, Yucatán México; 2 Campus Ciencias Biológicas y Agropecuarias. Universidad Autónoma de Yucatán, Merida Yucatán, México

**Keywords:** Leptospirosis, abortion spontaneous, Leptospira

## Abstract

**Introduction:**

Leptospirosis is a zoonotic disease affecting mainly to low income human population. Acute leptospiral infection during pregnancy has been associated with spontaneous abortion and fetal death during the first trimester and the abortion may occur as consequence of systemic failure.

**Objective:**

To estimate the frequency of *Leptospira interrogans* infection in women with spontaneous abortion in the state of Yucatan, Mexico.

**Methods:**

A cross sectional study on women with spontaneous abortion was conducted. Serum samples were tested for Leptospirosis by the microaglutination test, to estimate the frequency of the infecting serovar. The indirect ELISA IgM was used to detect recent infection by *L. interrogans*. DNA was extracted from paraffin-embedded tissue of placenta for PCR detection of *L. interrogans*.

**Results:**

Overall frequency of infection with *L. interrogans* in the 81 women with abortion was 13.6%. Five of the 12 serovars evaluated were found and included. Two of the 11 women with abortion and positive to microaglutination test were also positive to the ELISA IgM test. None samples were positive for PCR *Leptospira *diagnosis.

**Conclusion:**

two women could be associated with spontaneous abortion due to leptospirosis, because they showed antibodies against* L. interrogans* in the microaglutination test and ELISA IgM assays. Differences between regions were found with respect to the prevalences of lesptospirosis.

## Abstract (es)

**Introducción:**

Leptospirosis es una enfermedad zoonótica que afecta principalmente la población humana de bajos recursos. Infección aguda por leptospirosis durante el embarazo se ha asociado con aborto espontáneo y muerte fetal durante el primer trimestre del embarazo.

**Objetivo:**

Estimar la frecuencia de infección por *Leptospira interrogans* en mujeres con aborto espontáneo en el estado de Yucatán, México.

**Métodos:**

Se efectuó un estudio transversal en 81 mujeres con aborto espontáneo. La prueba de referencia para Leptospirosis, prueba de microaglutinación, se utilizó para estimar la frecuencia de la serovar infectante. El ELISA IgM indirecto se utilizó para detectar infección reciente por *L. interrogans.* Se extrajo ADN a partir de tejido embebido en parafina de placenta para la detección de *L. interrogans* por PCR.

**Resultados:**

La frecuencia global de la infección con *L. interrogans* en 81 mujeres con aborto, fue del 13.6%. Se encontró cinco de 12 serovares de *Leptospira*. Dos de las 11 mujeres con aborto espontáneo y positivo a microaglutinación también fueron positivas a la prueba ELISA IgM. Ninguna muestra fue positiva hacia el diagnóstico PCR de *Leptospira.*

**Conclusion:**

Dos mujeres podrían estar asociados con el aborto espontáneo debido a la leptospirosis, porque mostraron anticuerpos contra *L. interrogans* en la microaglutinación y ensayos de ELISA IgM. Diferencias entre regiones fueron encontradas con respecto a las prevalencias de leptospirosis.

## Introduction

Leptospirosis is a zoonotic disease affecting mainly to low income human population. The infection is caused by the spirochete *Leptospira interrogans* that comprises more than 200 serological variants in 23 groups. Its incidence is associated with social, cultural, occupational and climatic factors, and it is a worldwide spread disease of public concern because it may cause human death. The non-specific clinical presentation of the disease and the difficulty of its diagnosis in laboratories cause an underestimation of case numbers and its incidence. In humans, the incidence rate in some regions might be as high as 975 cases per 100,000 inhabitants 1.

Spontaneous abortion is one of the most common complications occurring during the first trimester of pregnancy, and 1 in 4 women experience an abortion during their reproductive career 2. Early pregnancy loss is defined as the end of pregnancy before 20 weeks of gestation or with a fetal weight of below 500 g. The main causes of abortion are genetic, immunologic, anatomical abnormalities, hematologic defects or endocrine or environmental causes as well as infection with several pathogens 3. Acute leptospiral infection during pregnancy has been associated with spontaneous abortion and fetal death during the first trimester and the abortion may occur as consequence of systemic failure 4-6 However, it is not known yet if abortion and fetal death occur as a direct or indirect effect of infection.

Leptospirosis in Mexico was first demonstrated in the state of Yucatan in 1920 7, and since then, different studies in humans and animal reservoirs confirm its endemic condition in Yucatan where it has been reported a 14.2% prevalence 8,9. However, the presence of *L. interrogans *in women with abortion has not been shown. The detection of leptospirosis in pregnant women and the fetuses may help the differential diagnosis of abortions, and generate information about the prevention of the disease. 

The objective of this study was to estimate the frequency of *Leptospira interrogans* infection in women with spontaneous abortion in two hospitals in the state of Yucatan, Mexico.

## Materials and Methods

A cross sectional study in women with spontaneous abortion was carried out at the Hospital Comunitario in the City of Ticul (January to June 2008) and at the Hospital Materno Infantil in City of Merida (February to June 2009), both located in the state of Yucatan, Mexico. Protocol was approved by the IRB of Universidad Autonoma de Yucatan. 

### Sampling method

Serum samples were obtained from a previous study on Toxoplasmosis conducted in a Hospital in which a high frequency of abortions (120 women over 7 mo in 600 pregnancies in 2007) was reported 10. Only 81 samples from the 100 women with spontaneous abortion examined from June 2008 to May 2009 were used. Forty-five samples (serum and placental tissue) corresponded to Hospital Comunitario of Ticul and 36 to the Hospital Materno Infantil of Merida. Sera were kept in a freezer at -70º C at the Laboratory of Infectious and Parasite Diseases of the Faculty of Medicine of the University of Yucatan. In addition, 40 paraffin inclusion tissue samples from placenta of aborted women coming from both hospitals were studied. 

### Microagglutination test (MAT)

The reference test for Leptospirosis, microaglutination test (MAT), was used to estimate the frequency of the infecting serovar. MAT was carried out according to the WHO 11 and Haake *et al* 12. Twelve of the known prevalent serovars in Yucatan were used as antigen on MAT. They were Pomona, Canicola, Hardjo, Tarassovi, Panama, Icterohaemorrhagiae, Grippotyphosa, Pyrogenes, Bratislava, Australis, Wolffi and Autumnalis 8,9. The microagglutination was observed with the aid of a Leica DM1000 microscope with a dark field condenser. A titer ≥100 was used as the cut-off value. All titers above this value were considered as evidence of exposure. When a cross-reaction was observed in a sample, the serovar with the highest titer was considered as the infective one.

### Indirect immune-enzyme assay (ELISA IgM)

The indirect immune-enzyme assay (ELISA IgM) was used to detect recent infection by *L interrogans*. A microtiter plate with flat-wells (F8 Maxisorp Nunc-INMUNO MODULE) was prepared adsorbing a pool of 12 sonicated *L interrogans* serovars (10mg/ml diluted in carbonate buffer pH 9.6). Two percent of BSA solution was used as the blocking agent and PBS Tween 20 solutions as the washing solution. The secondary antibody was anti-µ-chain-specific peroxidase conjugate (SIGMA), and OPD was used as the developer. 2.5 M H_2_SO_4 _was used to stop the reaction. The OD was measured in a Benchmark plus microplate reader (Bio-Rad, Hercules CA) at 490 nm. The cut-point used was the mean of the negative samples plus two standard deviations.

### Sample DNA extraction and PCR detection of Leptospira interrogans

DNA was extracted from paraffin-embedded tissue of placenta. In brief, a 10 µm cut was placed in an Eppendorf tube, and 500 µL of xylene was added to the tube and incubated for 30 min at RT to remove paraffin. Then, xylene was rinsed with 100% and 75% alcohol for 30 min each. After the rehydration procedure, the tissue was washed with PBS. Following removal of PBS, 200 µL of lysing buffer (20 mg/mL proteinase K, 10 µL 1M Tris-HCl solution, 2 µL 0.5 M EDTA, 100 µL 10% SDS) was added to the tube with the clean tissue and incubated overnight at 52° C. After the incubation period, the tube was centrifuged at 12,000 g for 10 min to remove debris. Supernatant was transferred to a fresh 1.5 mL Eppendorf and the conventional phenol-chloroform DNA extraction procedure was followed. The resulting DNA pellet was resuspended in 50 µL of distilled water.

To detect *L interrogans,* a semi-nested PCR procedure for the amplification of the 16S RNA gene was adapted from Murgia *et al* 13. In this study, we used the primers Lepat 1 (5'-GAG TCT GGG ATA ACT TT-3'); Lepat 2 (5'-AGA AAT TTG TGC TAA TAC CGA ATG T-3') and L4 (5'-GAT TTT TCG GGT AAA GAT-3') and a PCR mix was prepared accordingly to the manufacturer (Eppendorf, Hamburgo, Alemania) to which 100 ng/µL of sample DNA and 0.5 mM of each primer was added. Running conditions were an initial cycle at 94º C for 5 min, 40 cycles at 94º C for 1 min, 47º C for x 1.5 min, 72º C for 2 min, following of a cycle at 72º C for 7 min and it kept at 4º C degrees to stop the protocol. Amplification was observed in a Bio-Rad (Hercules CA) photo-documentation imager.

### DNA internal control

Beta-actin internal control was used to detect if DNA samples was intact amplifying a 290 bp PCR fragment from exon III from beta-actin gene. Primer forward: 5'-ACC CAC ACTGTG CCC ATC TA-3' and primer reverse: 5'-CGG AAC CGC TCATTG CC-3' following the same protocol used for *L. interrogans* detection using 60° C as a melting temperature. 

### Statistical analysis

The data were analyzed by descriptive statistics and the association of hospital with *Leptospira* infection was determined chi-square test, using the SPSS^®^ package.

## Results

The range of age of the women with spontaneous abortion was 14 to 42 years, with mean and standard deviation of 24.7+7.6 years. The frequencies of women by age, parity and age at abortion are shown in Table 1. Most women aborted at 5-13 weeks of gestation.

**Table 1.** Frequency of women by age, parity and age of abortion.

Factor	Frequency	%
Age (years)		
14-19	22	27.2
20-34	50	61.7
≥35	9	11.1
Parity		
Primiparous	40	49.3
Multiparous	40	49.3
Age at abortion (weeks)		
≤4	8	9.9
5-8	27	33.4
9-13	30	37.0
14-17	9	11.0
18-20	5	6.2

The overall frequency of infection with *L. interrogans* in the 81 women with abortion, using the MAT technique, was 13.6%. Ten (12.3%) of the infected women were detected in the Hospital Comunitario of Ticul (n= 45) and one (1.3%) in the Hospital Materno Infantil of Mérida (n= 36). No co-infection with toxoplasmosis was detected on the positive samples to *L. interrogans*.

Five of the 12 serovars evaluated were found and included to Hardjo, Gryppotyphosa, Borincana, Bratislava and Cynopteri. The serovars with the greatest number of positive cases and the highest titers were Hardjo and Gryppotyphosa (Table 2). 

**Table 2.** Positivity by serogroup, serovar and antibody titers against *L. interrogans* in women with spontaneous abortion.

Serogroup	Serovar	Serogroup		Antibody titer		
		Positives	%	1:100	1:200	1:400
*sejroe*	*Hardjo*	5	45.5	2	2	1
*grippotyphosa*	*gryppotyphosa*	2	18.2	1		1
*hebdomadis*	*borincana*	2	18.2	2		
*australis*	*bratislava*	1	9.1	1		
*cynopteri*	*cynopteri*	1	9.1	1		
Percentage				63.6	18.2	18.2

Two of the 11 women with abortion and positive to MAT were also positive to the ELISA IgM test. Of those two cases, one was infected by serovar Hardjo and the other by serovar Grippotyphosa, both with titers of 1:400. 

None of the 45 samples of placental tissue, tested using the PCR assay with Lepat1 and Lepat2 primers, were positive in the Hospital Comunitario of Ticul. To test, whether or not the extracted DNA from the samples was intact, the samples re-analyzed by PCR using universal probes to the beta-actin internal control. It was found that none of the samples had intact DNA.

## Discussion

Of the 81 women with abortion the 20 to 34 years old group was the most frequent probably because it is the most common age of having babies in the state of Yucatan (Table 1).

Most of the abortions occurred in the first stage of pregnancy (before the 12 weeks of pregnancy), a value similar to that reported in the guide for the diagnosis and treatment of spontaneous abortions by the Secretaria de Salud de Mexico 14 and in Chile 15.

The positivity to the MAT on women with spontaneous abortion had a similar percentage (13.6%) to the values found in open mixed populations (14.1, 14.0 and 14.2%) by Vado *et al*. 8, also in Yucatan. The most frequent serovar found here, was Hardjo (associated to cattle) followed by Gryppotyphosa and Bratislava (associated with wild animals and pigs, respectively), which have been reported in humans and domestic animals of Yucatan on previous studies 8,9. In Australia, Faine and Adler 4 reported a mortal case of intrauterine infection by *L. hardjo*, which was identified in the placenta and fetal tissues of a 30 weeks old fetus. In our study, two women were positive to ELISA IgM and MAT, one reacting to the Hardjo and the other to Gryppotyphosa serovars, in both cases with antibodies titers of 1:400, which suggest that these two women were coursing a recent or acute *Leptospira* infection. However, because leptospira were not found in the placental tissue, they cannot directly be associated with the main cause of abortion. The WHO 11 reported that leptospirosis during pregnancy may lead to abortion, fetal death, stillbirth or congenital leptospirosis in women depending of the period of pregnancy, but only a few of such cases have been reported.

A higher frequency of positive Leptospirosis cases was found in the rural Hospital Comunitario of Ticul as compared to the Hospital Materno-Infantil located in an urban area. These results agree with those of other authors 8,16, who observed that rural areas are at a highest risk of leptospirosis. This probably associated to a higher prevalence of Leptospirosis in animals (cattle, pigs and dogs) and contact of people with them, in the south of Yucatan, where the Hospital Comunitario of Ticul is located 9. However, no information of patient exposition to animals was collected at the time of sampling collection during the toxoplasmosis study. Gainder *et al*. 17, reported a case of Leptospirosis in a 20 years old nulliparous women (26 weeks pregnant) highly exposed to farm animals and rats, whereas Dadhwal *et al*. 18, reported a leptospirosis case of a 36 weeks pregnant women. Baytur *et al*. 19, on the other hand, reported a case in a nulliparous 26 weeks pregnant women exposed to sheep. 

## Conclusion

*Leptospira interrogans* represents a treat to pregnant women as evidenced in this study in which two women showed antibodies against* L. interrogans* in the MAT and ELISA IgM assays. Differences between regions were found with respect to the prevalences of leptospirosis and demonstrate the need of educational programs on rural areas to prevent transmission of the disease. The differential diagnosis of leptospirosis in women with abortions may contribute to better prevention programs against such disease including continuing education on zoonotic infections such as leptospirosis to physicians on rural hospitals.
